# Supplementary material for: Differentially CTCF-Binding Sites in Cattle Rumen Tissue during Weaning
Source: Int J Mol Sci. 2022 Aug 13;23(16):9070. doi: 10.3390/ijms23169070 (PMC9408924; doi:10.3390/ijms23169070)
Supplement: Supplementary file 1 [file ijms-23-09070-s001.zip › Supplementary Figures.pdf]

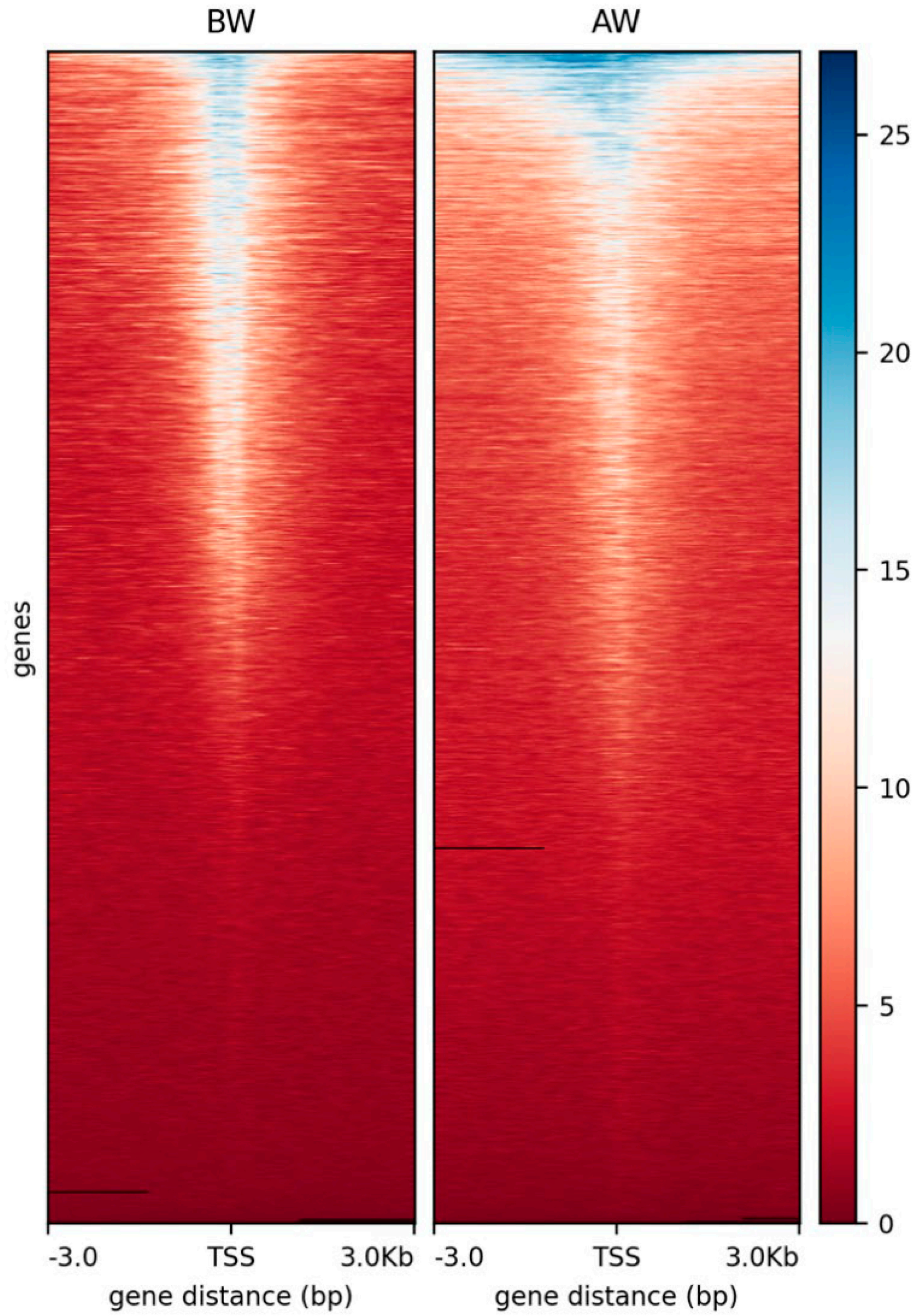


**Figure S1.** Heatmap profile of CTCF peaks relative to the transcription start sites (TSS) considering a ± 3 kb region for each pool with two samples each (considering chromosomes 1–29). Blue color intensity reflects the level of peak enrichment, and black lines are missing data. BW: before weaning. AW: after weaning.

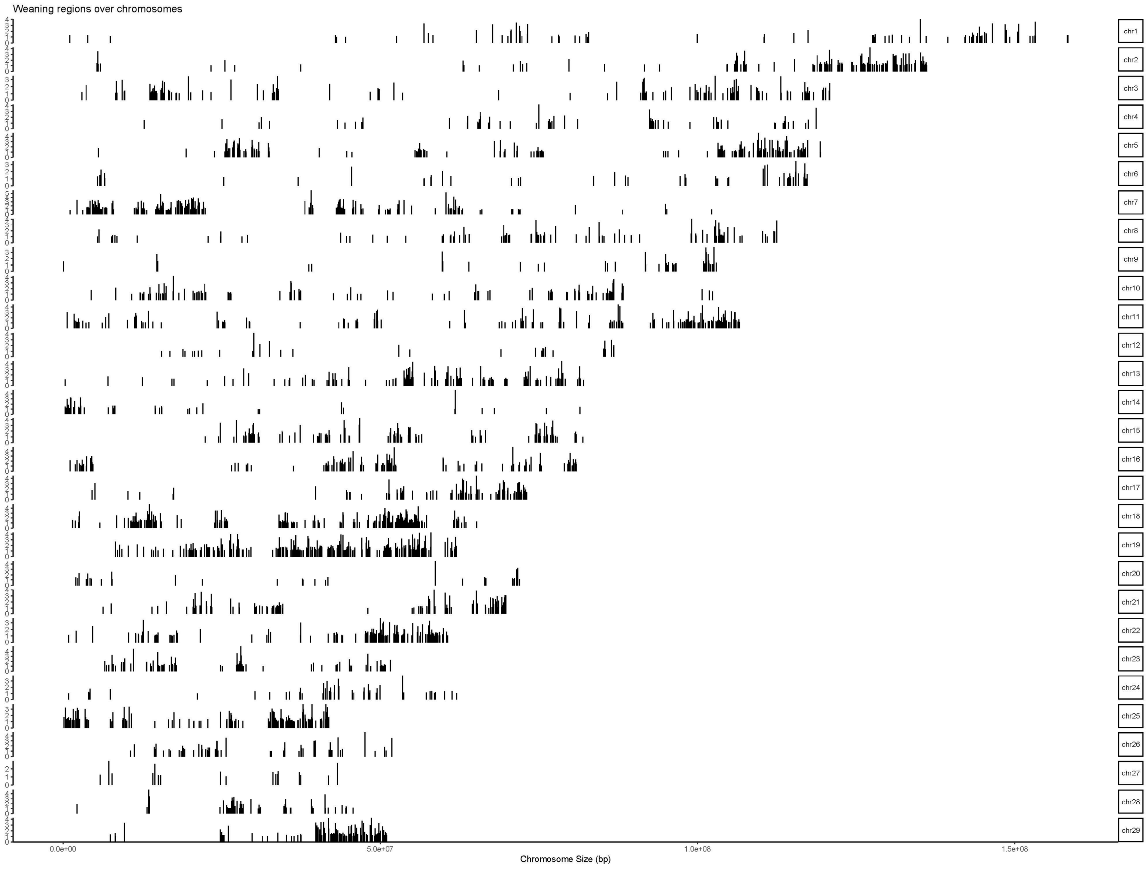


**Figure S2.** Chromosomal distribution of 7401 differentially CTCF-binding sites after weaning (considering bovine chromosomes 1–29).

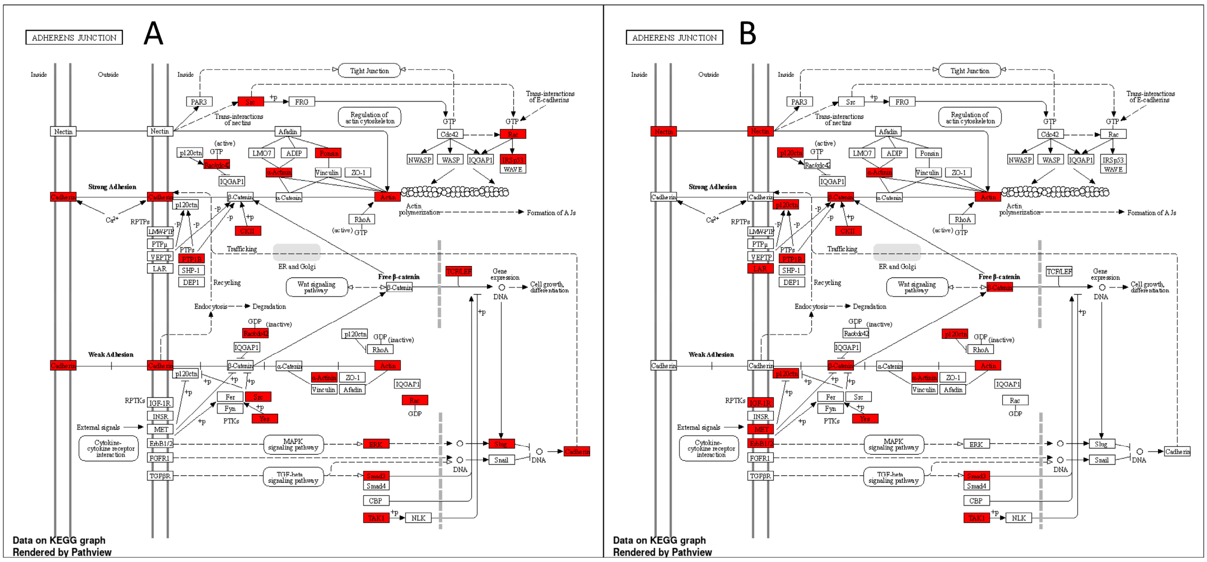


**Figure S3.** KEGG adherens junction pathway obtained from the (**A**) induced and (**B**) repressed differentially CTCF-binding sites (DCBS). Genes from the DCBS are highlighted in red.

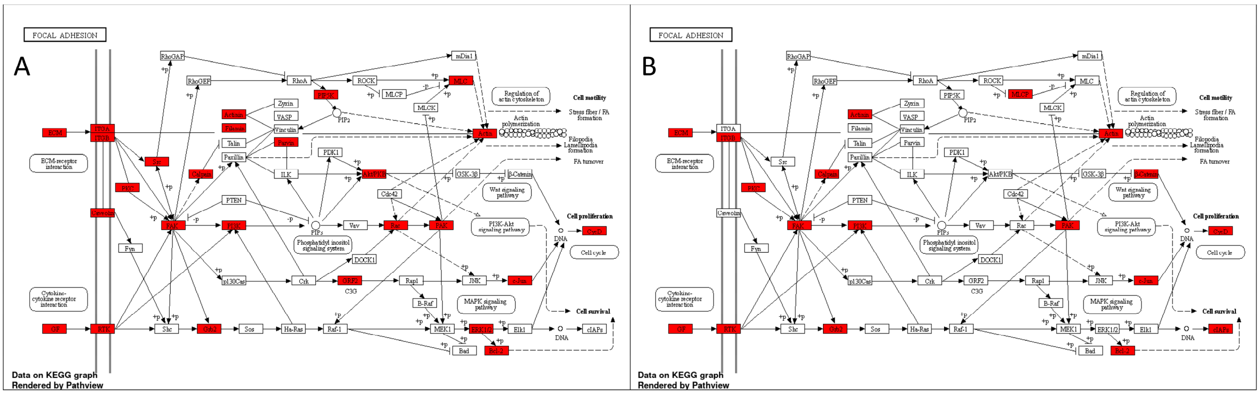


**Figure S4.** KEGG focal adhesion pathway obtained from the (**A**) induced and (**B**) repressed differentially CTCF-binding sites (DCBS). Genes from the DCBS are highlighted in red.

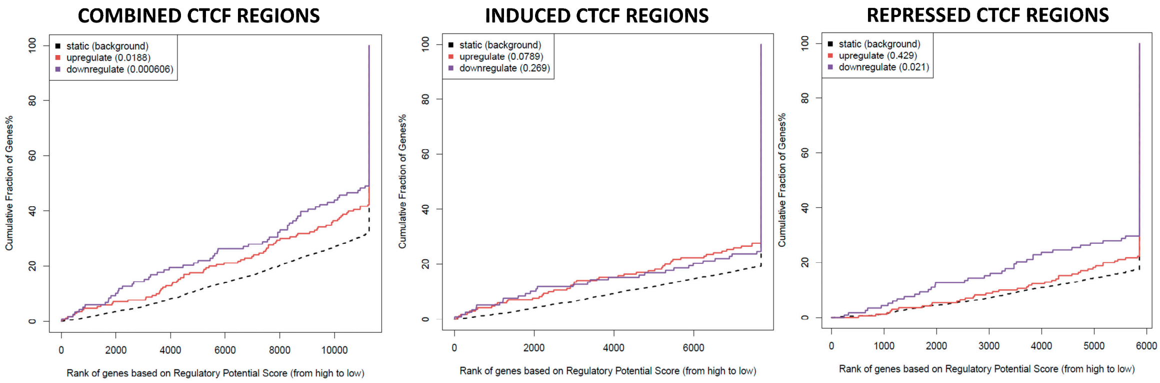


**Figure S5.** BETA activating and repressive function prediction of differentially CTCF-binding sites. The red line represents the up-regulated genes, the purple line represents the down-regulated genes, and the dashed line represents background genes with no differentially expressed genes. The *p*-values (inside the box) represent the significance of the difference between the up/down groups compared with the background genes by the Kolmogorov-Smirnov test.

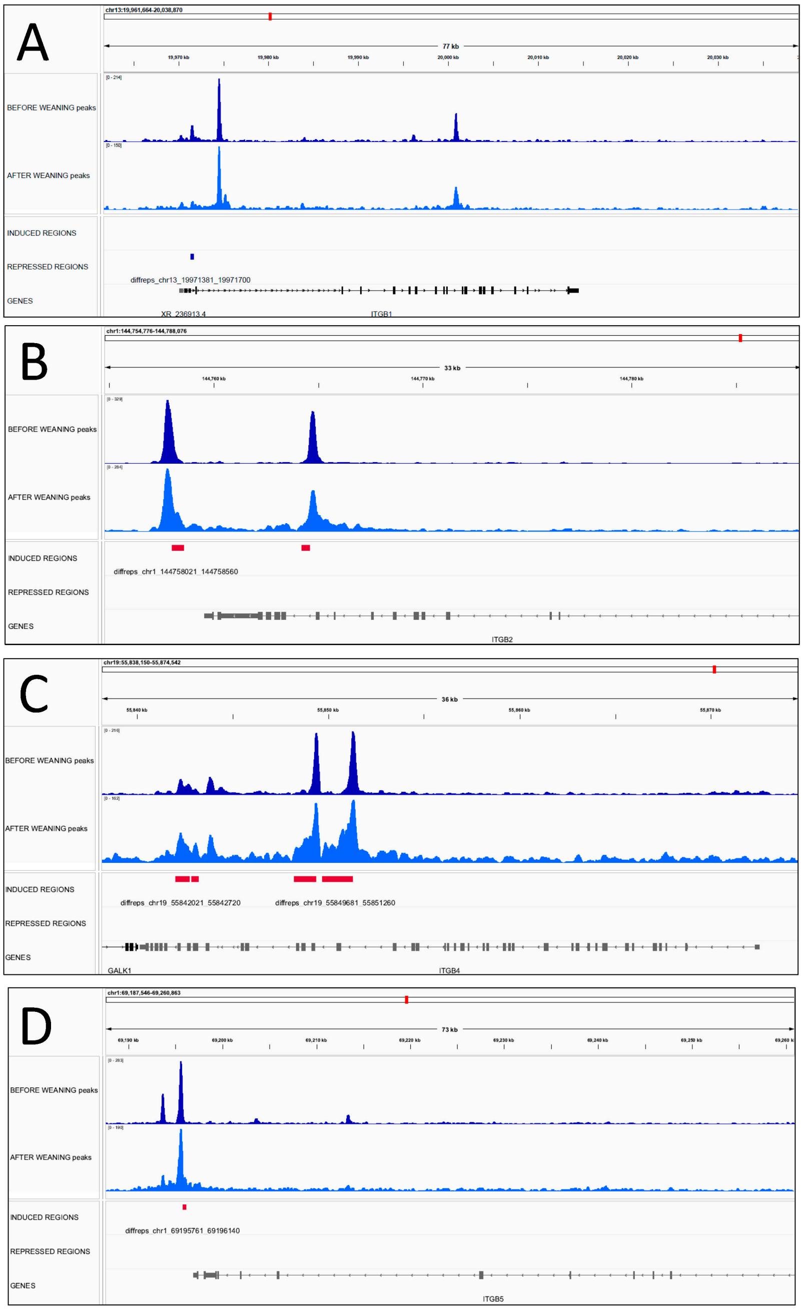


**Figure S6.** Integrative genomics viewer (IGV) screenshot of CTCF peaks (BW and AW) and the induced and repressed differentially CTCF-binding sites after weaning for the (**A**) *ITGB1* gene, (**B**) *ITGB2* gene, (**C**) *ITGB4* gene, and (**D**) *ITGB5* gene. The X-axis represents the chromosomal location with the size bar given in kb.

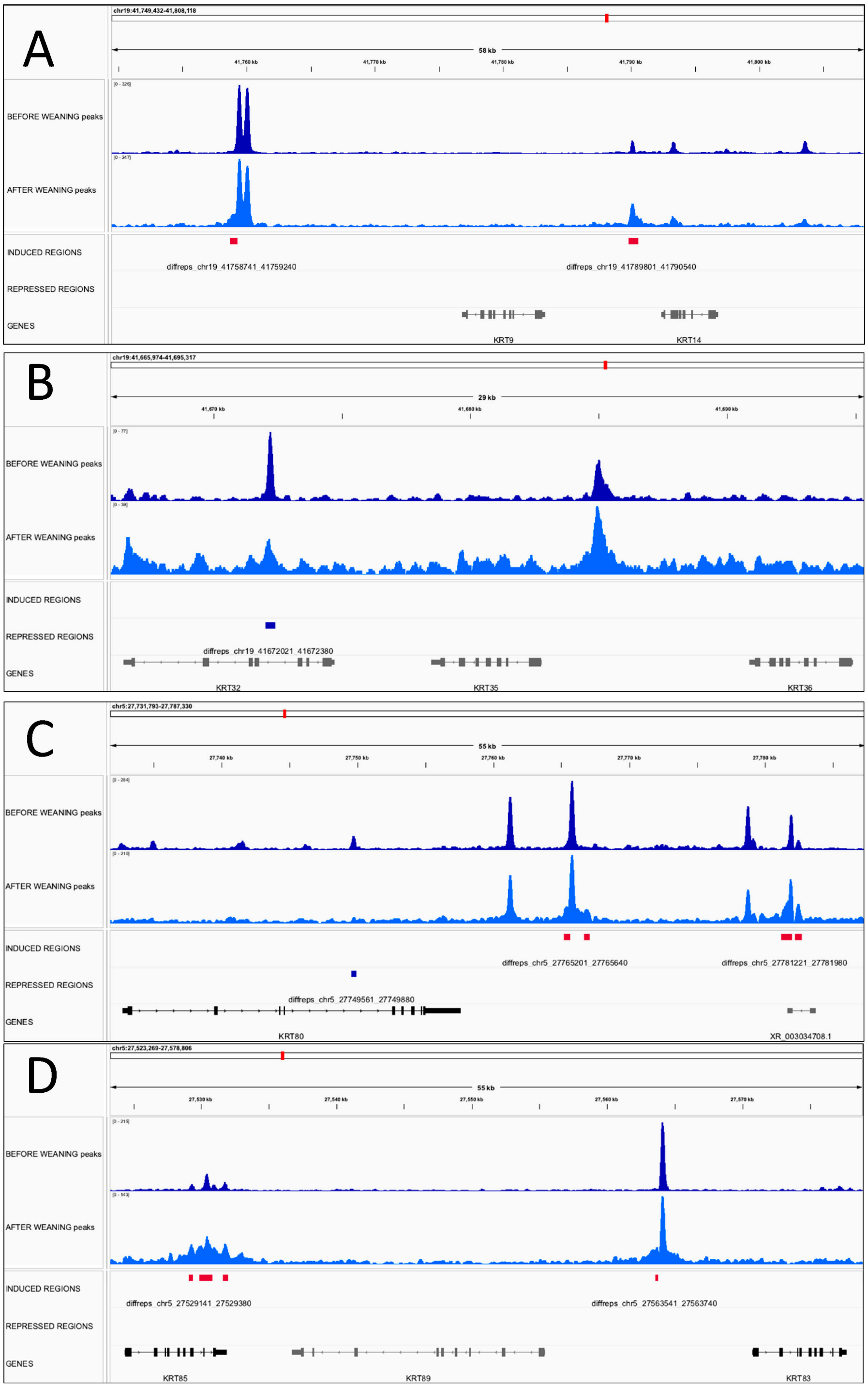


**Figure S7.** Integrative genomics viewer (IGV) screenshot of CTCF peaks (BW and AW) and the induced and repressed differentially CTCF-binding sites after weaning for the *keratin* genes - (**A**) *KRT9* and *KRT14*, (**B**) *KRT32*, *KRT35*, and *KRT36*, (**C**) *KRT80*, and (**D**) *KRT83*, *KRT85*, and *KRT89* genes. The X-axis represents the chromosomal location with the size bar given in kb.
